# Transient Global Amnesia following Neural and Cardiac Angiography May Be Related to Ischemia

**DOI:** 10.1155/2016/2821765

**Published:** 2016-06-21

**Authors:** Hongzhou Duan, Liang Li, Yang Zhang, Jiayong Zhang, Ming Chen, Shengde Bao

**Affiliations:** ^1^Neurosurgical Department, Peking University First Hospital, No. 8 Xishiku Street, Xicheng District, Beijing 100034, China; ^2^Cardiology Department, Peking University First Hospital, No. 8 Xishiku Street, Xicheng District, Beijing 100034, China

## Abstract

*Introduction*. Transient global amnesia (TGA) following angiography is rare, and the pathogenesis has not been illustrated clearly till now. The aim of this research is to explore the pathogenesis of TGA following angiography by analyzing our data and reviewing the literature.* Methods*. We retrospectively studied 20836 cases with angiography in our hospital between 2007 and 2015 and found 9 cases with TGA following angiography. The data of these 9 cases were analyzed.* Results*. We found all 9 cases with TGA following neural angiography (5 in 4360) or cardiac angiography (4 in 8817) and no case with TGA following peripheral angiography (0 in 7659). Statistical difference was found when comparing the neural and cardiac angiography group with peripheral group (*p* = 0.022). Two cases with TGA were confirmed with small acute infarctions in hippocampus after angiography. This might be related to the microemboli which were rushed into vertebral artery following blood flow during neural angiography or cardiac angiography. There was no statistical difference when comparing the different approaches for angiography (*p* = 0.82) and different contrast agents (*p* = 0.619).* Conclusion*. Based on the positive findings of imaging study and our analysis, we speculate that ischemia in the medial temporal lobe with the involvement of the hippocampus might be an important reason of TGA following angiography.

## 1. Introduction

Transient global amnesia (TGA) is a sudden-onset clinical syndrome characterized by a loss of memory for recent events and an inability to retain new memories; the patient is usually perplexed and disoriented in time and in place but without impairment of consciousness and personal identity [1]. During the onset, the neurological examination is often negative except for memory loss. TGA usually lasts for several hours to one day, and patients always recover well without any neurological abnormality [2]. TGA following angiography is rare; there is no more than 30 cases reported till now. Although vasospasm, transient ischemic attack (TIA), migraine, and seizure have been suggested as possible causes, the aetiology of TGA following angiography is still unclear [3]. We retrospectively studied all angiography cases in our hospital between 2007 and 2015, and we found nine cases with TGA following angiography. Here we present these 9 cases and try to analyze the pathogenesis of TGA following angiography.

## 2. Material and Methods

### 2.1. Data Collection

Two patients suffered from TGA following neural angiography in our neurosurgical department recently, and we also heard about some similar cases after cardiac angiography when communicating with a cardiologist; this aroused our interest. So we reviewed all patients with percutaneous angiography in our hospital from January 2007 to July 2015 by using electronic medical record system and consultation record system. As all the patients with TGA or other neurological deficits after angiography need a consultation from neurologist or neurosurgeon, so we can pick out all the TGA patients by using the consultation record system.

Totally 20836 cases being performed with intra-arterial angiography between 2007 and 2015 were studied, including 4360 cases with neural angiography (cerebral or spinal angiography, angioplasty, and embolisation), 8817 cases with cardiac angiography (coronary angiography, angioplasty, and ventriculography), and 7659 cases with peripheral angiography (endovascular examination or therapy beneath the aortic arch, such as renal angiography, angioplasty, embolisation of tumor in abdominal cavity or pelvic cavity, lower extremities angiography, and angioplasty). Nine patients diagnosed as TGA according to Caplan criteria (modified by Hodges and Warlow) [4] were enrolled in this study. We excluded the cases of memory disturbance together with motor or sensory deficiency, consciousness disturbance, dysphasia, or other neurological deficiencies. We also excluded the cases with amnesia not reversible and lasting for more than 3 days.

The whole data of these patients were analyzed, including the kind, dose, and injecting pressure of contrast agent, the type, route, and approach of angiography, vessel condition of vertebral basilar artery and subclavian artery, and other information such as age, hypertension, heparinization, and duration of angiography. The clinical manifestation, the duration of TGA, the imaging study after onset, and the follow-up data were also studied and retrospectively analyzed.

In the procedure of all neural angiography, 5 Fr catheter was positioned in bilateral subclavian artery, vertebral artery, or carotid artery via common femoral artery; larger guiding catheters like 6 Fr or 8 Fr were used in angioplasty or endovascular therapy. The contrast medium used in vertebral artery angiography was 4 mL/s and 6 mL in total, while it was 4 mL/s and 8 mL in total in subclavian artery or carotid artery angiography. The injection pressure was 250 psi (pounds per square inch) by automatic high pressure injector in all patients. Systemic heparinization (heparin, 80 IU/Kg) was introduced before all neural angiography patients without cerebral hemorrhage. For the patients with subarachnoid hemorrhage or cerebral hematoma, 2000 IU heparin added into 500 mL flushing saline was used during the angiography.

In cardiac angiography or angioplasty patients, 5 Fr or 6 Fr catheter was placed in coronary arteries, ascending aorta, or cardiac ventricle via radial artery, brachial artery, or common femoral artery. Allen's test and evaluation of the subclavian artery by using ultrasound should be performed before transradial or brachial artery approach is used. The injective dose and pressure were manually controlled. Systemic heparinization was given to all patients. Ventriculography was performed in some patients with the injection pressure 300 psi and dosage of 30 mL in 2 seconds by automatic high pressure injector.

All peripheral angiography was performed via common femoral artery, and the procedures were all conducted beneath the aortic arc, including angiography or embolisation of tumors in abdominal or pelvic cavity, angiography, or angioplasty of renal artery or lower extremities. The angiographic catheter was not inserted to the arteries above aortic arc. The dose, injection, and pressure were manually controlled or controlled by automatic high pressure injector, depending on the angiographic vessels. Systemic heparinization was given in most of the patients.

All patients received adequate hydration during and after angiography to prevent hypoperfusion and to speed up the excretion of the contrasts. If TGA happened, consultation with neurologists or neurosurgeons was required and intensive care was given. Computed Tomography (CT) or Magnetic Resonance Imaging (MRI) was performed, and then the treatment was given according to the results of imaging study. Most patients received conservative therapy including antiplatelet drugs or observation. All the patients were required to be followed up by phone-call or outpatient clinic.

### 2.2. Statistical Analysis

Univariate analyses were performed using Fisher's exact probability test for categorical variables. Numerical data were expressed as the median. Analyses resulting in *p* values less than 0.05 were considered statistically significant. All statistical analyses were performed with SPSS version 12.0 (Peking University Health Science Center, China).

## 3. Results

In 20836 cases with intra-arterial angiography, nine patients with TGA following angiography were found, six males and 3 females. Age ranged from 43 to 76 years with mean age of 62.4 years (Table 1). Four patients had the past history of hypertension and 3 patients with diabetes and only one female patient with migraine which was supposed to be a risk factor of TGA [5]. No patient suffered previous TGA. In 4360 cases with neural angiography, 5 patients (0.11%) experienced TGA following angiography, and the incidence of TGA following cardiac angiography was 0.045% (4 in 8817 cases). No TGA patient in peripheral angiography group (*n* = 7659) was found. There was no statistical difference in TGA occurrence when comparing cardiac angiography group with peripheral angiography group (*p* = 0.062) and cardiac angiography group versus neural angiography group (*p* = 0.152). But statistical difference was found when comparing neural angiography group with peripheral angiography group (*p* = 0.003). When cardiac angiography and neural angiography groups were added together as one group, comparing with the peripheral angiography group, there was still statistical difference (*p* = 0.022) (Table 2).

Two approaches were used in these 9 patients, transfemoral artery approach in six patients and transradial artery approach in other three patients. In these 9 patients, there was no statistical difference in TGA occurrence between these two approaches (*p* = 0.82). Two kinds of contrast agent with three different concentrations were used in these patients. There was also no difference in the occurrence of TGA when comparing these two kinds of contrasts (*p* = 0.619). The doses of contrast agent were all less than 200 mL, and there was no difference in injection pressure between TGA patients and no TGA patients. Blood pressure in these 9 patients was all controlled well during angiography, although one patient was a little nervous and had anxiety. The processes of angiography in these 9 patients all went smoothly and lasted no more than 2 hours.

In considering that the posterior cerebral circulation involved in the distribution of injected contrast agent and that the procedure of advancing the catheter via the subclavian artery might be two risk factors of TGA, the data of the subclavian artery and vertebral artery was studied. In the four cardiac angiography patients with TGA, preoperative ultrasound examination showed three of them with mild to moderate atherosclerotic stenosis in right subclavian artery. In the five TGA patients following neural angiography, intraoperative angiography showed three of them with normal subclavian artery, vertebral artery, basilar artery, and posterior cerebral artery, while the other two patients suffered severe stenosis vertebral artery in V1 segment and with normal intracranial arteries. During the subclavian or vertebral angiography, there was no observable vessel spasm or dissection. Although two patients underwent vertebral angioplasty and stent implantation, the procedure went smoothly and there was no visible dissection or vessel occlusion.

**Figure 1:**
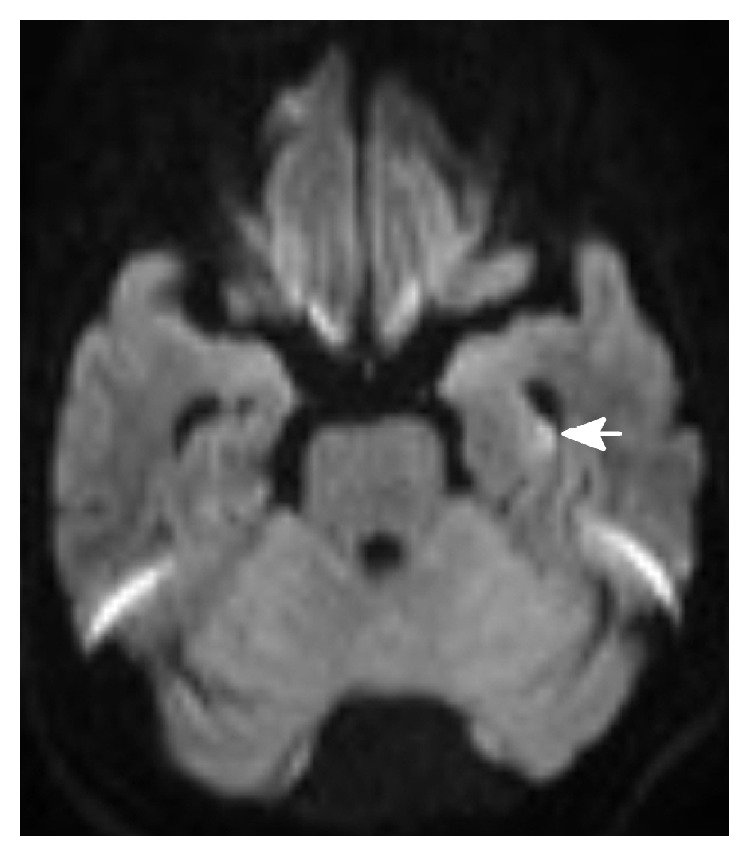
DWI of case 4 after TGA showing small acute infarctions (arrow) in left hippocampus.

**Figure 2:**
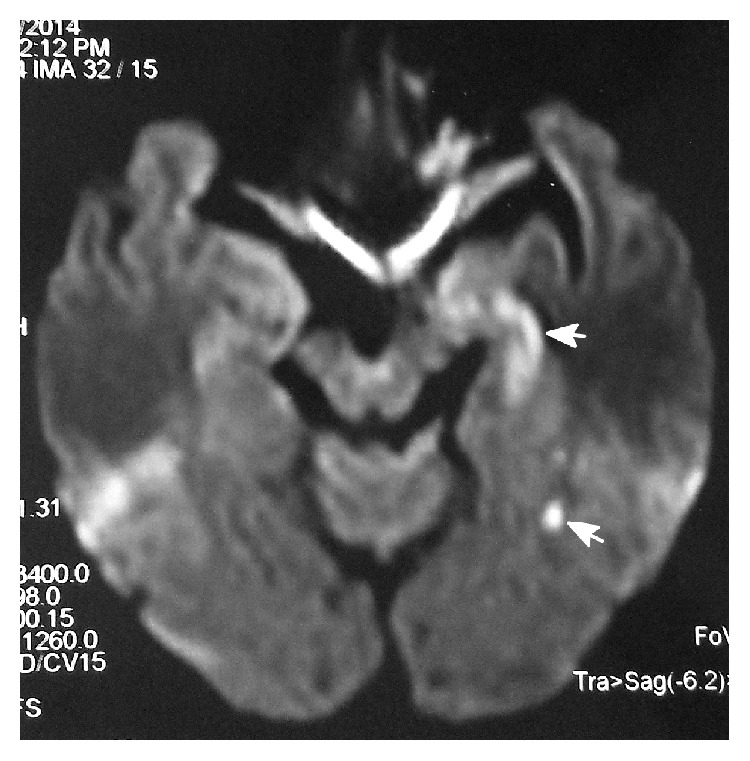
DWI of case 9 after TGA showing small acute infarctions (arrow) in left hippocampus and temporal lobe.

The clinical manifestation of TGA in these patients was similar. During or after angiography, patient appeared amnesic and perplexed: he/she was unable to recall events around the time of hospital admission. The retrograde memory for approximately several hours to several days was affected and the anterograde memory was also impaired. Sometimes the patient repeatedly asked why he was in the hospital, why he was scheduled to angiography, and where he was. The patients' attention was normal with Glasgow Coma Scale 15 scores. No other neurological deficiency was found. The average duration of TGA was 15.4 ± 7.47 hr. Five patients received CT scan after TGA, which did not show visible low density lesions (infarction) or hemorrhage. Four patients received MRI examination including transverse T1, T2, and T2 flair, DWI images, and sagittal T1 image. MRI images in two patients were normal without high density lesion in DWI. Two patients were confirmed with small acute infarctions (DWI) in left hippocampus or temporal lobe (Figures 1 and 2). Conservative therapy including intensive observation and/or antiplatelet drugs was given. All patients recovered well without any neurological deficit and discharged soon, and 4 patients were followed up with MRI which were all normal.

## 4. Discussion

TGA is considered a benign disorder as memory deficits resolve completely and do not lead to long-term sequelae. TGA following angiography is rarely reported. The pathogenesis has been a matter of long-standing debate among researchers. Many possible causes (ischemia, epileptic seizures, vasospasm, or a disturbance of venous hemodynamics) have been hypothesized. However, to date there is no convictive explanation [5]. Although we also have no sufficient evidence to illuminate the exact pathogenesis of TGA following angiography, we want to reasoning the cause based on our cases and the reports in the literature.

When we encountered TGA following angiography for the first time, we have speculated several reasons. As the initial clinical manifestation was retrograde amnesia, just like the patients with cerebral concussion after traumatic brain injury, we supposed that there might be a transient concussion injury in the hippocampus region due to the “water hammer effect” caused by excessive pressure or rapid injection during the vertebral artery angiography. As reported in the literature, in up to 70% of reported TGA cases, a precipitating event—mainly described as physical or emotional stress—is present [5]. But there were some differences of amnesia between traumatic brain injury and TGA. In TGA patients, both retrograde memory and anterograde memory were all impaired, which indicated that there might be functional or organic damage in the memory system including hippocampus. We reviewed our TGA patients, and all blood pressure was controlled well during the operation. And there were also 4 TGA patients following cardiac angiography in our study, which could not be explained by the “water hammer effect,” because the catheter had not been placed into vertebral artery and the contrast was injected manually with low injection pressure during angiography.

In the literature, some researchers attributed TGA to the contrast agent because of its chemotoxicity, osmolality, and viscosity which would destroy the blood brain barrier and damage the neurons, especially the neurons in hippocampus [6]. Although nonionic contrast has replaced the ionic contrast nowadays, there were still several reports about TGA following angiography with different nonionic contrasts [6–14]. But interestingly, we found that there was no report of TGA after intravenous contrast injection such as enhanced CT examination in PubMed. Here we have no sufficient evidence to deny the contrast as one of the reasons of TGA, but we think that there might be other more convictive reasons.

In our research, we found that all cases were followed by neural or cardiac angiography, and there was no TGA case following peripheral angiography, which was the same as in the literature. The type of angiography was correlated with the occurrence of TGA when comparing the neural and cardiac angiography group with peripheral group (*p* = 0.022). We speculate that ischemic embolism theory might be responsible for this phenomenon. During neural angiography or cardiac angiography via radial or brachial artery, the catheter should be advanced through subclavian artery; if some small intima or atherosclerotic plaque was rubbed down, it will be flushed away and may embolise small intracranial vessels. And during the process of cardiac angiography via femoral artery, although the catheter might not be placed in the subclavian artery, catheter-induced emboli, particulate matter, or air embolus in the contrast agent would also flush into the cerebral circulation by blood flow and result in embolism. However, in peripheral angiography group, both the subclavian artery and the cerebral blood flow were not involved during angiography, so embolism in posterior cerebral circulation and TGA will not happen.

High resolution imaging studies have demonstrated diffusion-weighted imaging lesions selectively in the CA-1 region of the hippocampus in TGA patients. By using diffusion-weighted MRI 24–48 h after a TGA episode, small dot-like lesions have been detected in the hippocampus [15]. The involvement of the mesial temporal structures explains the clinicoanatomic correlation between the location of the signal change, the procedure, and symptoms of memory loss. As our report, Graff-Radford et al. [3] and Hahn et al. [1] reported 2 cases of TGA following angiography with small infarction of hippocampus or hypoperfusion of temporal lobe, which indicated that an ischemic mechanism might play a great role in the course of TGA following angiography.

There are many mechanisms which result in ischemia, such as vasospasm induced by contrast media or catheter, hypoperfusion during angiography, embolism by dislodged atherosclerotic plaque, catheter-induced emboli, or particulate matter in the contrast agent. Jackson et al. described a series of six patients who experienced TGA or cortical blindness during selective vertebral angiography and attributed it to vertebral arterial spasm induced by injection of contrast above body temperature [2]. In our 9 cases, two patients were confirmed with small acute infarctions in left hippocampus and the medial temporal lobe and other 7 cases without positive finding in image study. In these 7 cases, 5 patients received CT scan which cannot show a small acute infarction as clearly as MRI, and two other patients who received MRI examination also showed negative findings. We speculate that there might be two reasons responsible for these two patients without positive MRI findings. One reason is that these 2 patients might have suffered from TIA in hippocampus and temporal lobe areas, which has no positive finding in MRI examination. The other reason is that dot-like hyperintense lesions are usually found in the lateral aspect of the hippocampus on DWI in the subacute phase after TGA, which usually appear 24 to 72 hours after onset [16]. The MRI examinations taken in these two patients were both less than 13 hours after onset, so the positive findings might be missed.

The weakness of our study is that not all of these patients received MRI after TGA, and only two patients were confirmed with fresh embolism in hippocampus. However, after a meticulous analyzation of the 20836 cases in our hospital and reviewing the reports in the literature, we infer that the ischemic hypothesis might explain most of TGA cases following angiography.

## 5. Conclusion

In conclusion, although many mechanisms were mentioned in the literature, we speculate that ischemia in the medial temporal lobe with the involvement of the hippocampus caused by dislodged atherosclerotic plaques, catheter-induced emboli, particulate matter, or air embolus in contrast agent might be an important reason of TGA following angiography.

## Figures and Tables

**Table 1:** Data of the nine patients with TGA following angiography.

Pt	G/A	Preoperative diagnosis	Past history	Angiography					TGA			Follow-up (month)
				Type	Approach	Condition of VA and rSCA	Contrast agent	Dose (ml)	Length (hr)	Examination after TGA	Results of imagine	
1	M/57	Aneurysm	HT	NA	TFA	Normal	Iohexol 300 mgL/mL	85	18	MRI, MRA (12 hr)	Negative	6 months, normal MRI
2	F/43	Ependymoma	Normal	NA	TFA	Normal	Iohexol 300 mgL/mL	72	24	CT (18 hr)	Negative	—
3	M/56	Spinal AVF	Fracture	NA	TFA	Normal	Iohexol 300 mgL/mL	98	8	CT (3 hr)	Negative	—
4	M/73	Stroke	HT, DM	NA, VA angioplasty	TFA	Severe stenosis in VA	Iodixanol 270 mgL/mL	164	20	MRI (26 hr) Ultrasound	Small acute infarction in hippocampus	6 months, normal MRI
5	M/60	VA stenosis	HT, HL	NA, VA angioplasty	TFA	Severe stenosis in VA	Iohexol 300 mgL/mL	125	12	CT (6 hr)	Negative	12 months, normal MRI
6	M/76	AMI	HT, DM	CA, angioplasty	TRA	Moderate stenosis in rSCA	Iodixanol 270 mgL/mL	140	20	MRI (5 hr)	Negative	—
7	F/58	Stable angina	HL	CA	TRA	Normal	Iohexol 350 mgL/mL	56	3	CT (24 hr)	Negative	—
8	F/67	Follow-up of CABG	Migraine	CA	TFA	Mild AS in rSCA	Iohexol 350 mgL/mL	75	24	CT (3 hr)	Negative	8 months, normal MRI
9	M/72	Stable angina	DM	CA	TRA	Mild AS in rSCA	Iohexol 350 mgL/mL	66	10	MRI (28 hr)	Small acute infarction in hippocampus	—

AMI: acute myocardial infarction; AS: atherosclerosis; AVF: arteriovenous fistula; CA: cardiac angiography; CABG: coronary artery bypass graft; CT: computed tomography; DM: diabetes mellitus; F: female; G/A: gender/age; HL: hyperlipemia; HT: hypertension; M: male; MRI: magnetic resonance imaging; NA: neural angiography; Pt: patient; rSCA: right subclavian artery; TFA: transfemoral approach; TGA: transient global amnesia; TRA: transradial approach; VA: vertebral artery; —: lost follow-up.

**Table 2:** Comparing TGA patients in different types of angiography, approach, and contrast.

	Total patients (*n*)	TGA patients (*n*)	*χ* ^2^ and *p* value
*Type of angiography*			
Neural angiography	4360	5	Total *χ* ^2^ = 8.48, *p* = 0.014^*∗*^ N/P, *χ* ^2^ = 8.787, *p* = 0.003^*∗*^ C/P, *χ* ^2^ = 3.475, *p* = 0.062 N/C, *χ* ^2^ = 2.053, *p* = 0.152 N + C/P, *χ* ^2^ = 5.233, *p* = 0.022^*∗*^
Cardiac angiography	8817	4	
Peripheral angiography	7659	0	

*Type of approach*			
Transfemoral approach	13126	6	TFA/TRA, *χ* ^2^ = 0.052, *p* = 0.82
Transradial approach	7707	3	

*Type of contrast agent*			
Iohexol	17476	7	Iohexol/Iodixanol, *χ* ^2^ = 0.247, *p* = 0.619
Iodixanol	3360	2	

TGA: transient global amnesia; N: neural angiography; C: cardiac angiography; P: peripheral angiography; TFA: transfemoral approach; TRA: transradial approach.  ^*∗*^ *p* < 0.05, statistical difference.
